# Supplementary material for: Causal Relationship between Aging and Anorexia Nervosa: A White-Matter-Microstructure-Mediated Mendelian Randomization Analysis
Source: Biomedicines. 2024 Aug 16;12(8):1874. doi: 10.3390/biomedicines12081874 (PMC11351342; doi:10.3390/biomedicines12081874)
Supplement: Supplementary file 1 [file biomedicines-12-01874-s001.zip › Supplementary Figure/Figure S4/Singe SNP plot of MR analysis for TL to ubm-b-2076.pdf]

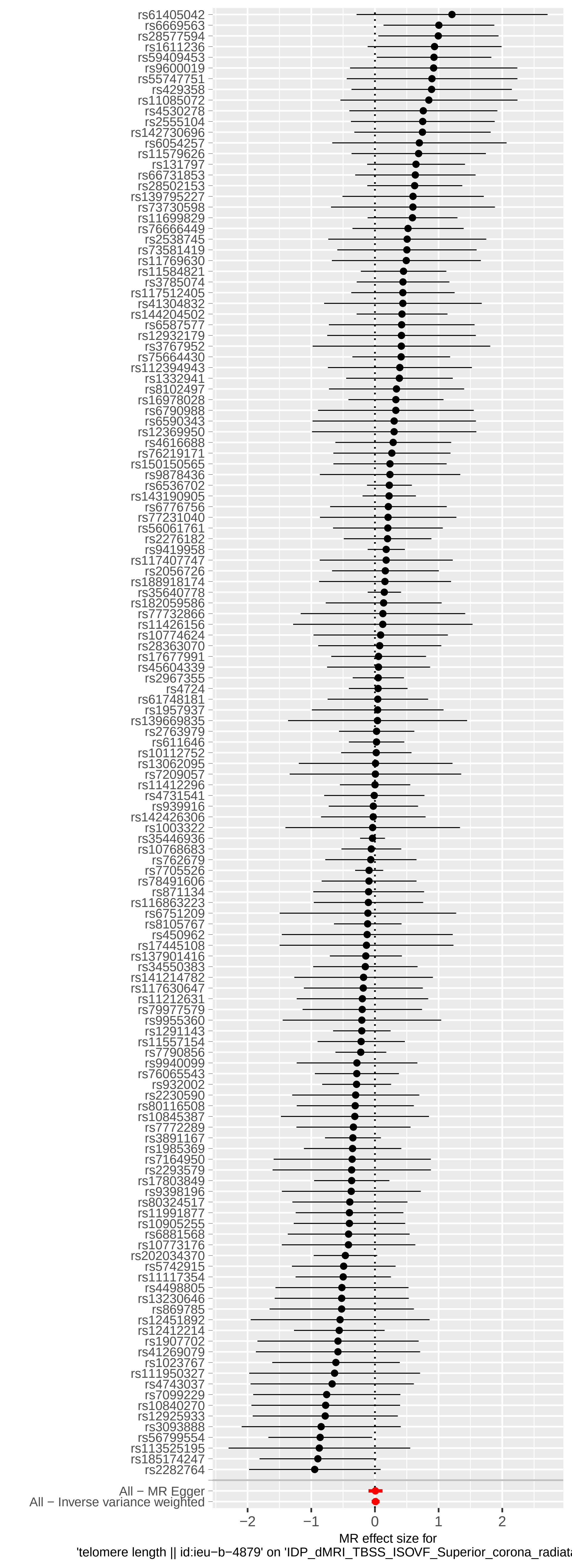
rs61405042
rs6669563
rs28577594
rs1611236
rs59409453
rs9600019
rs55747751
rs429358
rs11085072
rs4530278
rs2555104
rs142730696
rs6054257
rs11579626
rs131797
rs66731853
rs28502153
rs139795227
rs73730598
rs11699829
rs76666449
rs2538745
rs73581419
rs11769630
rs11584821
rs3785074
rs117512405
rs41304832
rs144204502
rs6587577
rs12932179
rs3767952
rs75664430
rs112394943
rs1332941
rs8102497
rs16978028
rs6790988
rs6590343
rs12369950
rs4616688
rs76219171
rs150150565
rs9878436
rs6536702
rs143190905
rs6776756
rs77231040
rs56061761
rs2276182
rs9419958
rs117407747
rs2056726
rs188918174
rs35640778
rs182059586
rs77732866
rs11426156
rs10774624
rs28363070
rs17677991
rs45604339
rs2967355
rs4724
rs61748181
rs1957937
rs139669835
rs2763979
rs611646
rs10112752
rs13062095
rs7209057
rs11412296
rs4731541
rs939916
rs142426306
rs1003322
rs35446936
rs10768683
rs762679
rs7705526
rs78491606
rs871134
rs116863223
rs6751209
rs8105767
rs450962
rs17445108
rs137901416
rs34550383
rs141214782
rs117630647
rs11212631
rs79977579
rs9955360
rs1291143
rs11557154
rs7790856
rs9940099
rs76065543
rs932002
rs2230590
rs80116508
rs10845387
rs7772289
rs3891167
rs1985369
rs7164950
rs2293579
rs17803849
rs9398196
rs80324517
rs11991877
rs10905255
rs6881568
rs10773176
rs202034370
rs5742915
rs11117354
rs4498805
rs13230646
rs869785
rs12451892
rs12412214
rs1907702
rs41269079
rs1023767
rs111950327
rs4743037
rs7099229
rs10840270
rs12925933
rs3093888
rs56799554
rs113525195
rs185174247
rs2282764
All − MR Egger
All − Inverse variance weighted
−2
−1
0
1
2
MR effect size for
'telomere length || id:ieu−b−4879' on 'IDP_dMRI_TBSS_ISOVF_Superior_corona_radiat
